# Clinical Utility of Small Extracellular Vesicles as Liquid Biopsy for Oral Mucosal Disease Diagnostics: Emerging Perspectives

**DOI:** 10.3390/diagnostics16071044

**Published:** 2026-03-30

**Authors:** Olawande Funmilola Adebayo, Dada Oluwaseyi Temilola, Foluso John Owotade, Manogari Chetty

**Affiliations:** 1 Department of Oral Medicine and Oral Pathology, Obafemi Awolowo University Teaching Hospitals Complex, Ile-Ife 220282, Nigeria; fowotade@oauife.edu.ng; 2 Department of Craniofacial Biology, Pathology and Radiology, University of the Western Cape, Cape Town 7535, South Africa; dtemilola@uwc.ac.za (D.O.T.); mchetty@uwc.ac.za (M.C.)

**Keywords:** oral mucosal diseases, sEVs, liquid biopsy, diagnosis, biomarkers

## Abstract

Some diseases affecting the oral mucosa can be life-threatening and/or associated with life-threatening complications. Conventional diagnostic methods for most oral mucosal diseases are usually employed at a fully established disease state. All these peculiarities usually result in late diagnosis, poor prognosis, poor treatment outcomes, and reduced overall survival rates, hence the need for novel methods for the early detection of these disease conditions. Small extracellular vesicle (sEV)-based diagnosis carries great potential for early diagnosis of oral mucosal diseases, as sEVs reflect the physiological status of their parent cells. sEVs are also widely distributed in body fluids, which helps overcome the problem of inaccessibility in sample or specimen collection in some cases. Furthermore, the composition of sEVs can be used as diagnostic biomarkers for several disease conditions, including oral mucosal diseases. This review critically examines the emerging role of sEVs-derived biomarkers from saliva and blood in the diagnosis of some oral mucosal diseases, such as hand, foot, and mouth disease (HFMD), oral lichen planus (OLP), oral leukoplakia (OL), and oral squamous cell carcinoma (OSCC). It also discusses the need for the validation and standardization of the potential sEV-derived diagnostic biomarkers of these oral mucosal diseases for clinical application.

## 1. Introduction

The oral mucosa consists of multiple layers of epithelium, known as stratified squamous epithelium, lying on a basement membrane supported by connective tissue [1,2]. It lines almost the entire oral cavity and extends from the lip to the mucosa of the pharynx, excluding the teeth [3]. It serves several functions, such as the protection of underlying structures, such as bone, muscles, blood vessels, and nerves. It subserves sensory function for thermal, touch, pain, and taste perception, and it facilitates the secretion of chemical substances through saliva and gingival crevicular fluid [2]. Other important functions include the following: absorption of medications and other substances; elimination of toxic and harmful substances from the system; and digestion through taste receptors present on different oral mucosal sites [1]. In some cases, the oral mucosa serves as an indicator of the status of an individual’s systemic health [4].

According to the WHO, diseases affecting the oral mucosa can be classified into three categories on the basis of clinical characteristics and etiology, which include inflammatory, reactive, and neoplastic disorders [5]. These disease conditions impair the function of the oral mucosa, which negatively affects the oral health and general health of individuals affected, hence resulting in a reduction in quality of life [2,5]. Considering the impact of oral mucosal diseases on the overall well-being of patients, there is a need for improvement in their treatment outcomes. Good treatment outcomes and prognosis of oral mucosal disease largely depend on early and accurate diagnosis, especially in cases of oral potentially malignant diseases and malignant disease conditions.

Tissue-based biopsy is still the gold standard investigation method for diagnosing most oral mucosal lesions. However, it has drawbacks, including delayed diagnosis due to late clinical presentation, potential sampling inaccuracies arising from lesion heterogeneity, and challenges in obtaining representative specimens from anatomically inaccessible sites [6]. Another challenge of tissue biopsy is the negative impact of patients’ perception of postoperative pain, discomfort, and healing duration on their oral health [7]. These limitations call for a highly sensitive, specific, less invasive, and convenient method of diagnosis.

Liquid biopsy has recently emerged as a promising alternative to overcome these limitations [6,8]. This approach is based on the application of biomarkers from circulating molecules such as circulating tumor cells, circulating tumor DNA, cell-free DNA, and extracellular vesicles in body fluids.

Extracellular vesicles (EVs) are small lipid bilayer-bound vesicles released from all body cells into the extracellular environment. Usually, EVs can be classified into two main subtypes based on their characteristics and biogenesis pathway: ectosomes and small extracellular vesicles (sEVs). Ectosomes, generated by cytoplasmic membrane budding, typically measure 100–1000 nm in diameter [9]. In contrast, sEVs, also known as “exosomes”, range from 30 to 150 nm and are released by the fusion of multivesicular bodies (MVBs) with the plasma membrane [9]. SEVs are nanosized extracellular vesicles that are widely distributed in almost all body fluids [10,11], and they contain biomolecules such as lipids, proteins, and nucleic acids, which reflect the physiological status of their cell of origin [12,13]. Specific biomolecules are upregulated, downregulated, or absent in disease conditions, hence their application as diagnostic biomarkers [13]. They also serve as prognosticators, predictors of progression or malignant transformation, metastatic biomarkers, and therapeutic tools for disease conditions [14,15].

The diagnostic potential of sEV biomarkers in specific oral mucosal diseases has been increasingly recognized. In 2011, Sharma et al. observed the increased size (mean (SD) in OSCC patients: 98.3 (4.6 nm); mean (SD) in healthy controls: 67.4 (2.9 nm)) and increased aggregation and number of salivary sEVs in oral squamous cell carcinoma (OSCC)-affected individuals compared with healthy controls [16]. The study also reported an increased level of circulating sEVs in the plasma of the patients with OSCC relative to healthy individuals [16], and these findings were corroborated by Zlotorgoski et al. in 2016 [17].

This narrative review article discusses the potential application of saliva and blood-derived sEV biomarkers in the diagnosis of common oral mucosa diseases such as hand, foot, and mouth disease (HFMD), oral lichen planus (OLP), oral leukoplakia (OL), and oral squamous cell carcinoma (OSCC). It also highlights previous studies carried out on saliva and blood sEVs conducted in the field of oral mucosal diseases (Table 1).

## 2. Literature Search Methods

A comprehensive literature search was conducted in May 2025 using the Google Scholar and PubMed databases. The search terms included combinations of the following keywords: *small extracellular vesicles, extracellular vesicles, exosomes, microvesicles, oral mucosal diseases, hand foot and mouth diseases, oral lichen planus, oral leukoplakia, oral squamous cell carcinoma, OSCC biomarkers, miRNA, circRNA, and lncRNA*. Only peer-reviewed articles that investigated the diagnostic potential of small extracellular vesicles and related vesicle subtypes in oral mucosal disease were included. The reference lists of key publications were also screened to identify additional relevant studies.

### 2.1. Biogenesis, Composition, and Biological Functions of sEVs

#### 2.1.1. Biogenesis

Small extracellular vesicles (sEVs) are a type of extracellular vesicles (EVs) found in biological fluids [32,33,34]. They were initially discovered about 50 years ago by Wolf et al., who called them “platelet dust” [33]. EVs are commonly classified into three groups based on size and mechanism of secretion: the smallest being sEVs, which are about 30 to 150 nm in size; microvesicles, which are intermediate in size, ranging from 100 to 1000 nm; and apoptotic bodies, the largest, measuring about 500 nm to 5000 nm [32,34].

sEVs are endosomal in origin [34,35]. Although the process of their formation is not completely understood, it can be summarized into three major steps: (i) early endosome formation; (ii) maturation of the early endosome into multivesicular bodies (MVBs); and (iii) the fusion of the MVBs with the cell’s plasma membrane to release sEVs [35,36].

Early endosomes are formed by the inward budding of the plasma membranes of the cells [35,36]. Early endosomes eventually mature into MVBs by the inward budding of their own limiting membranes. MVBs play key roles in endocytic function and the transport of cell material [35,36]. A specific MVB can either form an sEV by fusing with the plasma membrane of the cell or be degraded by a lysosome [35,36]. The outcome of the MVBs is dependent on their cholesterol levels [35]. Those rich in cholesterol eventually form sEVs, while those lacking cholesterol follow the other route [35].

The biogenesis of sEVs can comprise an endosomal sorting complex required for transport (ESCRT)-dependent or -independent mechanisms [35]. The ESCRT-independent regulatory mechanisms of sEVs biogenesis include the sphingomyelinase enzyme [37], tetraspanin proteins [32,34,35,38], and simple integral membrane proteins of the lysosome/late endosome [32] (SIMPLE) pathways, amongst others. Certain growth factors that regulate the formation of MVBs for sEV production according to their needs have also been described [34].

#### 2.1.2. sEVs Composition

The molecular composition of sEVs is determined by their biogenesis, parent cell type, and status or treatment [32,34,39]. An sEV consists of a lipid bilayer containing biomolecules, such as proteins; lipids; and nucleic acids, such as DNA, RNA, miRNA, and other non-coding RNA [40,41].

Lipids are the most important contents of the sEVs, contributing to biogenesis, the maintenance of their structure and form, and homeostasis regulation in the cell [42,43]. Different classes of lipids are found in sEVs. They include sphingolipids, such as ceramide, gangliosides, and sphingomyelins; phospholipids such as phosphatidylserine, phosphatidylethanolamines, phosphatidylcholine, lysophosphatidylcholine, and phosphatidylinositols; diacylglycerols; and cholesterol [44]. Ceramides facilitate the sorting of cargo into MVBs [39]; lysobisphosphatidic acid enhances MVB’s budding and sEV development [39,43]; sphingomyelin, phosphatidylcholine, and phosphatidylserine are involved in the fate and differentiation of sEVs [39,42]; lysophosphatidylcholine and arachidonic acid are responsible for membrane curvature [39]; cholesterol is crucial in the regulation of sEV secretion [39].

Proteins are also vital elements found on the membrane and interior of sEVs [45]. They participate in biogenesis, membrane fusion, and the release of sEVs. They are also involved in disease development [45]. sEV proteins can be broadly classified into two categories: common and specific proteins [46].

The common proteins in sEVs can be further sub-categorized into four types, which include the following: (a) membrane fusion and transport-related proteins such as Ras-associated binding-GTPases, annexin, and heat shock proteins (HSPs), which are important in membrane fusion and sEV release; (b) ESCRT proteins such as tumor ALG-2-interacting protein X (ALIX), susceptibility gene (TSG 101), and vacuolar protein sorting-associated protein 4 (VPS4), which are key players in sEVs biogenesis; (c) four transmembrane cross-linked proteins, such as intercellular adhesion molecule 1 (ICAM-1) and tetraspanin-8 (TSPAN8), which are important in intercellular communication, ESCRT-independent pathways of biogenesis, and the potential utilization of sEVs as therapeutic tools; and (d) cytoskeletal proteins, such as integrins, actin, myosin, tubulin, cofilin, etc. [45,46,47].

The specific sEV proteins are based on the parent cell type and condition [42,48]. Different specific proteins are found in sEVs derived from different biological fluids in the same disease condition. In addition, sEVs derived from the same biological fluid, for example, plasma, express differing specific proteins in different disease conditions. The specificity forms the basis for the application of sEV proteins as biomarkers of disease diagnosis, prognosis, and progression.

Nucleic acids such as DNA and RNAs, which include messenger RNA (mRNA), microRNA (miRNA), ribosomal RNA (rRNA), P-element-induced wimpy testis-interacting RNA (piRNA), transfer RNA (tRNA), long non-coding RNA (lncRNA), small nucleolar RNA, and circular RNA (circRNA), are also present in sEVs [6,39,49]. The nucleic acid components reflect the homeostatic and functional status of the parent cells [50].

MicroRNAs are the most abundant RNAs in sEVs [51]. They regulate key cell processes, such as cell development, angiogenesis, hematopoiesis, exocytosis, and oncogenesis [52,53]. They are intercellular communication molecules and hold promise as predictive diagnostic and prognostic markers in disease conditions, particularly cancers [52,53]. They can also be used as therapeutic tools.

#### 2.1.3. Biological Functions of sEVs

Like the constituents, the biological function of sEVs is dependent on the parent cell and its status at the time of sEV formation [40].

**Figure 1:**
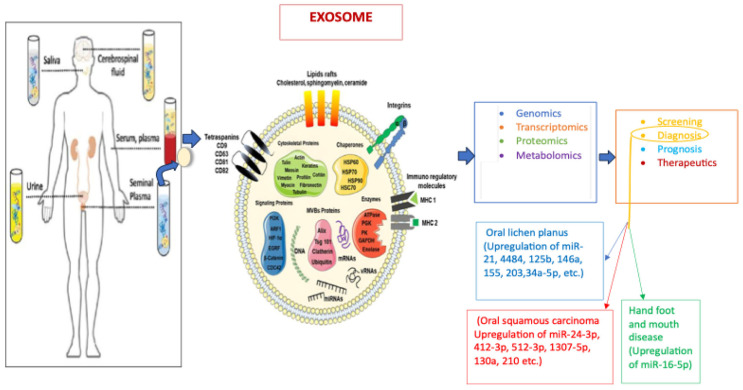
sEVs in body fluids and their downstream applications.

sEVs were thought to be only waste bags; however, they have been found to perform various biological roles in health and disease [40]. They are able to perform these various roles as a result of their ability to transfer biomolecules between cells, therefore influencing physiological and pathological processes in disease conditions such as malignancies, infections, autoimmune diseases, and neurodegenerative conditions [40]. Beyond cellular waste disposal, sEVs are involved in intercellular signaling and communication; immune function, such as antigen presentation [54] and the differentiation of regulatory T cells or myeloid cells for immune suppression [55]; and the development and differentiation of stem cells [56]. They also play an important role in physiological processes, such as coagulation, inflammation, apoptosis, and cellular homeostasis [40]. Other biologic functions of sEVs include tumor progression by the promotion of angiogenesis [57]; tumor cell migration in metastases [58], and pathogen spread [59]. The relevance of sEVs across multiple omics fields, such as genomics, proteomics, and metabolomics, has also been explored (Figure 1).

### 2.2. Isolation and Characterization of sEVs

#### 2.2.1. Isolation of sEVs

sEVs are present in virtually all body fluids, including blood, saliva, urine, semen, cerebrospinal fluid, synovial fluid, breast milk, epididymal fluid, amniotic fluid, effusions from malignancies, etc., and they can be isolated from these sources [60,61]. Several sEV isolation techniques have been described, each based on the distinct physical and biochemical properties of sEVs [62].

Isolation methods based on physical properties, such as particle size and weight, include ultracentrifugation; size-based isolation techniques include ultrafiltration, exclusion chromatography, and polymer-based precipitation [63]. The immunoaffinity capture method uses the biochemical properties of sEVs, such as proteins, for isolation, whereas the microfluidics isolation technique integrates both physical and biochemical principles [63]. Commercial isolation kits employing one or a combination of the basic isolation methods are also available. The examples include the following: Exoquick^TM^; Exosome Precipitation Solution; Total Exosome Isolation kit; RIBO^TM^ Exosome Isolation Reagent; Exo-spin; ME kit; ExoQuick Plus and Exo-Flow; etc. [64,65].

Ultrafiltration and precipitation methods of isolation are simple procedures allowing high-throughput processing [63], in contrast to ultracentrifugation and size exclusion chromatography methods, which are complex procedures that require expensive instruments [63,66]. The immunoaffinity capture method is also expensive [63]. Despite these challenges, size-exclusion chromatography, immunoaffinity capture, and microfluidics-based methods typically produce sEVs of higher purity and with better preserved structural integrity than other approaches [61,66,67,68]. Precipitation methods, however, tend to yield larger quantities of sEVs [66,67].

#### 2.2.2. Characterization of sEVs

Three forms of sEV characterization have been described. They include the following: qualitative, quantitative, and single-vesicle characterization [63].

Qualitative characterization involves the identification of sEVs by the validation of their proteins, lipids, and nucleic acid markers [63,69]. Quantitative characterization assesses the outcome, yield, and purity of the sEVs isolated in terms of their biomolecules, such as proteins, lipids, and nucleic acids [63,69]. The single-vesicle characterization focuses on the analysis of size, structure, and chemical composition of individual sEVs [63].

In biomedical research, quantitative characterization is most commonly performed. It entails the total sEV count using nanoparticle tracking analysis [70]; morphology characterization using transmission electron microscopy [71]; particle size characterization with the dynamic light scattering method [72]; total sEV protein count by a bicinchoninic acid (BCA) assay [71]; specific sEV protein measurements using Western blotting; ELISA; mass spectrometry and flow cytometry; nucleic acid quantification by microarrays; next-generation sequencing; and quantitative polymerase chain reaction (qPCR) [73].

### 2.3. sEVs as Biomarkers for Diagnosis of Oral Mucosal Diseases

#### 2.3.1. Hand, Foot, and Mouth Disease (HFMD)

HFMD is an acute viral illness characterized by fever and oral ulcers preceded by vesicular rashes on the hands and feet [74,75]. Its symptoms were first reported in 1957 in Toronto [76]. HFMD usually affects children under five years of age [74], although it can also occur in immunocompromised or immunosuppressed adults. The disease is caused by human Enteroviruses of the *Picornaviridae* family, such as EVA-71, CVA16, CVA6, CVA10, CVB1-5, etc. [74,77]. Of all the enterovirus species implicated in HFMD, EVA-71 and CVA16 account for greater than 70% of reported outbreaks [74,75].

HFMD is usually associated with spontaneous resolution within a few days with no complications. However, there could be fatal cardiopulmonary and neurological complications in rare instances [75]. Conventionally, the diagnosis of HFMD relies on clinical history, examination, and investigation, including pathogen identification via reverse transcription polymerase chain reaction (RT-PCR), virus isolation, and neutralizing antibody testing [78]. The use of the sEV miRNA as a potential diagnostic tool in HFMD was demonstrated in a study by Jia et al. [18]. The study observed the upregulation of miR-16-5p (by 5.98 fold in mild HFMD and 10.31 fold in extremely severe HFMD patients) and the downregulation of miR-150-3p and miR-671-5p in the serum sEVs of children with HFMD compared with healthy children [6,18,36].

More studies are required to validate the dysregulation of these sEV miRNAs for their application in the diagnosis of HFMD. The confirmation of this finding should also be carried out with salivary sEVs.

#### 2.3.2. Oral Lichen Planus (OLP)

OLP is a chronic inflammatory and immune-mediated disorder of the oral mucosa [36,79,80]. It affects 0.5–2% of the general population [79]. Lichen planus can affect other body sites, such as the skin and mucosae of the genitals, esophagus, and eyes [80]. Although the exact cause of OLP is unknown, some risk factors have been identified [79,80]. They include the following: genetic predisposition, such as inheritance of HLA genes (HLA A3, A5, A7, A11, A26, A28, B7, DR1, DR10, and DRW9); immune dysregulation; infections, such as viral infections (human papilloma virus (HPV), Epstein–Barr virus (EBV), hepatitis C virus (HCV), HIV, and HHV6) and bacterial infection with Helicobacter pylori; hormonal factors; stress; food allergies; and inflammatory bowel disease [80,81].

Different clinical variants of OLP exist, and they have been broadly classified into three types based on their appearance [36,79,82]. There is the hyperkeratotic variant, which consists of the reticulate, papular, and plaque/verrucous types; the erosive/ulcerative type, which is made up of erosive and erythematous/atrophic forms; and the rare bullous OLP [79]. OLP is usually asymptomatic but may be symptomatic in some cases presenting pain and discomfort, dysgeusia, xerostomia, burning sensation, and psychological distress [79,80]. The WHO regards OLP as an oral potentially malignant disorder with about a 1–2% risk of malignant transformation [83]. Of all OLP types, the erosive forms carry the highest risk of transformation [84].

Diagnoses typically involve history, clinical examination, and investigation, such as biopsy for histopathology and direct immunofluorescence [36,79]. Studies have revealed the potential application of sEV biomarkers in the diagnosis of OLP. The upregulation and downregulation of certain salivary, blood, and tissue sEV microRNAs have been observed and can thus serve as diagnostic biomarkers of OLP.

Byun et al. observed an increased expression of miR-4484 in the salivary sEVs of patients with OLP compared with healthy controls [19]. Higher levels of miR-21 [20,21], miR-125b [21], miR-203 [21], and miR-15b [21], as well as lower levels of miR-125a [20], miR-27b [21], miR-146a [22], and miR-155 [22,85], have also been demonstrated in the salivary sEVs of OLP patients compared to healthy controls. In addition, the upregulation of salivary sEV miR-31 is noted in OLP patients with developmental anomalies but absent in those with no developmental anomalies [20,86]. 

Furthermore, Peng et al. found an upregulation of sEV miR-34a-5p and miR- 130b-5p and downregulation of miR-301b-3p in the plasma of patients with OLP when compared with healthy volunteers [23]. Yang et al. further described the induction of upregulation of chemokines, such as macrophage-induced protein (MIP)-1alpha/beta, IL-10, and IL-17 by T-lymphocyte-derived sEVs, which play a key role in the development of OLP [87]. These inflammatory chemokines may also serve as OLP diagnostic biomarkers.

#### 2.3.3. Oral Leukoplakia (OL)

OL is the most common oral potentially malignant disorder [88]. The WHO defines OL as a white patch of questionable risk, having excluded all other potential causes that carry no increased risk of carcinoma [89]. Although the precise etiology is not clear, strong risk factors include smoking; alcohol drinking; betel quid chewing, especially in the southeast region of the Asian continent; and some genetic factors [6,88,90,91].

Traditionally, the diagnosis of OL usually involves history, clinical assessment, and biopsy for histopathological examination. However, the great potential of OL to transform into malignancy necessitates early detection for surveillance and follow-up. sEV biomarkers carry the potential for the early diagnosis of oral leukoplakia [6].

Čema et al. reported increased levels of SolCD44 and total protein in salivary sEVs of patients with OL, with concentrations correlating positively with clinical severity [24]. These findings support the further exploration of sEV-derived biomarkers for early OL detection.

#### 2.3.4. Oral Squamous Cell Carcinoma

OSCC is the most common histologic variant of oral cancer, accounting for about 90% of cases. Oral cancer is still one of the most common cancers worldwide, with an annual incidence of over 389,000 cases [92]. GLOBOCAN has projected a 65% increase in the incidence of OSCC by 2050 [92,93]. OSCC usually affects individuals older than 50 years and more males than females. However, an increasing incidence is now observed in women and young people due to a shift in the practice of traditional risk factors [93,94]. Tobacco and alcohol consumption are known strong risk factors for OSCC. Other possible risk factors include the following: chronic oral inflammatory conditions, HPV infection, and a diet low in antioxidants [93,94,95].

Despite the recent advances in OSCC diagnosis and treatment, poor prognosis and low survival rates are still associated with the disease [96]. Early diagnosis is key to overcoming these challenges. The utility of sEV biomarkers can offer the early detection of OSCC, which will, in turn, improve the prognosis and survival rate of patients. Several sEV biomarkers for OSCC have been reported [97]. 

Some studies have observed upregulation in the size, number, concentration, and aggregation of salivary sEVs in OSCC patients compared with healthy individuals [16,17,25]. Moreover, Nakamichi et al., in 2021, found that protein cargo in saliva-derived sEVs is a promising diagnostic biomarker for OSCC [98]. The increased expression of protein CD63 and the reduced expression of CD81, 82, and 9 in OSCC patients, in comparison with healthy individuals, have also been noted in saliva-derived sEVs [17]. In addition, a pilot study by Bozyk et al. observed the expression of some sEV proteins, such as PSB7, AMER 3, and LOXL2, in the saliva of patients with OSCC, which are potential diagnostic biomarkers for the early detection of OSCC [12,97]. The potentiality of some serum sEV proteins, such as laminin 332 [99], APOA1 [100], CXCL7 [100], PF4V1 [100], and F13A1 [100], as diagnostic biomarkers of OSCC lymph node metastasis has also been reported.

Salivary microRNA dysregulation has also been demonstrated with higher levels of miR-412-3p [25], miR-512-3p [25], miR-24-3p [26], miR-31 [101], miR-27a-3p [25], miR-373-3p [25] miR-494-3p [25], miR-486-5p [27], and miR-1307-5p [28] and lower levels of miR-10b-5p [27] in OSCC patients compared to healthy controls [17]. Specific miRNAs, such as miR-302b-3p and miR-517b-3p, are only expressed in the salivary sEVs of OSCC patients [25]. 

The serum sEV-associated squamous cell carcinoma antigen (SCCA) has been identified as another potential biomarker. Although elevated in OSCC patients, its diagnostic performance improves markedly when combined with saponin-based extraction methods rather than conventional isolation techniques [31]. In addition, the upregulation of microRNAs, such as miR-210 [29] and miR-130a [30], has been observed in the plasma of OSCC patients relative to healthy individuals, highlighting additional candidates for early detection.

## 3. Limitations and Future Directions

Although sEV-based biomarkers hold considerable promise as diagnostic tools, their translation into clinical practice remains limited by several challenges. Biological variability among individuals; technical variability across isolation and characterization methods; insufficient large-scale clinical validation; and the absence of standardized protocols all undermine the reliability and reproducibility of sEV cargo profiles. These limitations collectively hinder the development of robust and generalizable diagnostic biomarkers.

The high cost of liquid biopsy technology poses an additional barrier, particularly in African countries and other low- and middle-income countries (LMICs) where healthcare budgets are constrained. Furthermore, the implementation of sEV-based diagnostics often requires specialized equipment and trained personnel, resources that may not be readily available in many LMIC settings.

Future research will need to prioritize the development of liquid biopsy-based biomarkers that are feasible, affordable, and scalable within resource-limited environments. Population-based studies are essential to address biological variability and facilitate the validation of population-specific biomarkers. Continued innovation in liquid biopsy technologies is required to reduce costs, minimize technical variation, and simplify operational requirements.

At the policy level, increased governmental investment in health infrastructure, laboratory capacity, and workforce training will be critical to support the integration of sEV-based diagnostics into routine clinical care. Large, multi-center studies employing rigorous methodological designs will also be crucial for validating and harmonizing sEV biomarkers to meet clinical implementation standards.

With sustained scientific, technological, and policy advancements, EV-based diagnostics have the potential to become a central component of precision oral medicine, enhancing early detection, improving diagnostic accuracy, enabling patient stratification, and ultimately contributing to better treatment outcomes.

## 4. Conclusions

The diagnostic potential of salivary and blood-derived sEVs in oral mucosal diseases represents a significant advancement in non-invasive biomarker research. Evidence across multiple studies demonstrates that sEV cargos, particularly microRNAs and specific proteins, undergo disease-specific modulation in conditions such as hand, foot, and mouth disease, oral lichen planus, oral leukoplakia, and oral squamous cell carcinoma. These molecular signatures offer promise not only for early detection but also for monitoring disease progression and therapeutic response.

## Figures and Tables

**Table 1:** Saliva and blood-derived sEVs studies and their findings in countries of the world.

Country	Type of Oral Mucosa Diseases	Source of Small Extracellular Vesicles	Analytical Techniques	Findings	Exosomal Biomarker	Sample Size	Author (Year)
China	Hand, foot, and mouth disease (HFMD)	Serum	Microarray method	Upregulation in HFMD patients compared with controls	miR-16-5p	54 (18 ESHFMD, 18 MHFMD, and 18 healthy controls)	Jia et al. 2014 [18]
				Downregulation in HFMD patients compared with controls	miR-150-3p and miR-671-5p		
South Korea	Oral lichen planus (OLP)	Saliva	miRNA microarray analysis and TaqMan quantitative polymerase chain reaction	Upregulation in OLP patients compared with controls	miR-4484	24 (16 OLP patients and 8 healthy controls)	Byun et al. 2015 [19]
Iran		Saliva	Quantitative RT-PCR	Upregulation in OLP patients compared with controls	miR-21	60 (30 OLP patients, 15 OSCC patients, and 15 healthy controls)	Mehdipour et al. 2018 [20]
				Downregulation in OLP patients compared with controls	miR-125a		
Italy		Saliva	Low-density microarray analysis and qRT-PCR	Upregulation in OLP patients compared with controls	miR-21, miR-125b, miR-203, and miR15b	A systematic review of 6 research articles	Stasio et al. 2019 [21]
				Down regulation in OLP patients compared with controls	miR-27b		
Iran			RT-qPCR	Upregulation in OLP patients compared with controls	miR-146a, miR-155	60 patients (15 patients each with and without dysplastic OLP, 15 OSCC patients, and 15 healthy controls)	Mehdipour et al. 2023 [22]
China		Plasma	Exosomal miRNA microarray analysis and quantitative real-time RT-PCR confirmation	Upregulation in OLP patients compared with controls	miR-34a-5p and miR- 130b-5p	30 (19 OLP patients and 11 age- and sex-matched healthy controls)	Peng et al. 2018 [23]
				Downregulation in OLP patients compared with controls	miR-301b-3p		
Latvia	Oral Leukoplakia (OL)	Saliva	OncAlert^®^ oral cancer rapid test	Increased level in OL patients compared to healthy controls and increasing level with severity	SolCD44 and total protein	70 (50 OL patients and 20 controls with benign lesions)	Čēma et al. 2021 [24]
USA	Oral squamous cell carcinoma (OSCC)	Saliva		Irregular in OSCC patients	Morphology of sEVs	10 (5 OSCC patients and 5 healthy volunteers)	Sharma et al. 2011 [16]
				Increased in OSCC patients compared with healthy controls	Size of sEVs		
				Increased in OSCC patients compared with healthy controls	Particle aggregation of sEVs		
Israel		Saliva	Transmission electron microscopy, atomic force microscopy (AFM), and nanoparticle tracking analysis (NTA)	Increased value in OSCC patients compared with controls	Morphology, size, and concentration of sEVs	61 (36 OSCC patients and 25 healthy individuals)	Zlotogorski- Hurvitz et al. 2016 [17]
			ELISA and Western blotting	Higher concentration in OSCC patients compared with healthy controls	CD 63		
				Lower concentration in OSCC patients compared with healthy controls	CD 9, 81, 82		
Italy		Saliva	qRT-PCR	Overexpression in OSCC patients compared with controls	miR-27a-3p, miR-373-3p, miR-494-3p	32 (21 OSCC patients and 11 healthy controls)	Gai et al. 2018 [25]
				Higher levels in OSCC patients compared with healthy individuals	miR-412-3p and miR-512-3p		
				Expressed only in OSCC patients	miR-302b-3p and miR-517b-3p		
China		Saliva	miRNA microarray analysis and qRT-PCR	Higher levels in OSCC patients compared with healthy individuals	miR-24-3p	8 (4 OSCC patients and 4 healthy controls)	He et al. 2020 [26]
Romania		Saliva	qRT-PCR	Higher levels in OSCC patients compared with healthy individuals	miR-486-5p	50 (25 OSCC patients and 25 healthy controls)	Faur et al. 2022 [27]
				Lower levels in OSCC patients compared with healthy individuals	miR-10b-5p		
India		Saliva	Real-time PCR	Overexpression in OSCC patients compared with controls	miR-1307-5p	17 (12 OSCC patients and 5 healthy controls)	Patel et al. 2022 [28]
Italy		Plasma	qRT-PCR	Overexpression in OSCC patients compared with controls	miR-210	44 (30 OSCC patients and 14 healthy controls)	Bigagli et al. 2022 [29]
China		Plasma	qRT-PCR	Higher in OSCC patients compared to controls	miR-130a	380 (184 OSCC patients and 196 healthy controls)	He et al. 2021 [30]
China		Serum		Increased expression in OSCC patients	Squamous cell carcinoma antigen (SCCA)	186 (73 OSCC patients and 113 healthy controls	Yang et al. 2022 [31]

ESHFMD: Extremely severe HFMD; MHFMD: mild HFMD; qRT-PCR: quantitative real-time PCR; ELISA: enzyme-linked immunosorbent assay.

## Data Availability

Data are contained within this article.
